# Fractures of the Cervix Femoris

**Published:** 1873-12

**Authors:** Sam’l Peters

**Affiliations:** Crescent, N. Y.


### Correspondence.

### Fractures of the Cervix Femoris.

MR. EDITOR:—*Buffalo Medical and Surgical Journal.*

A Correspondence in the Boston Medical Journal, Sept. 18th, 1873, signed "Rusticus," reminds me of corroborating facts which may be worth noting,

The writer referred to, in speaking on the subject of splints for fractures says: "I remember that my old master used to tell us that Sir Astley Cooper said that if he should have a fracture of the cervix femoris, he would have his leg put on a pillow, in the most comfortable position possible, and after the lapse of a few weeks, would get up and go about, as well as he could with a crutch."

During a practice of about thirty years, I have treated in this manner substantially six cases of this fracture, and all of them successfully, so that, with a shortening of from $2\frac{1}{4}$ to 4 inches, the patients were able to walk very comfortably: The difference in length being made up by a cork sole or some similar contrivance. At the solicitation of friends, or counciling Surgeons, I have treated two cases with cord, pulley and weight, and, so far as my experience goes, old people bear such restraining appliances badly, causing an unfavorable reaction upon the repairative process. Indeed all things being equal, I much prefer the treatment, especially for old people, recommended by Sir Astley Cooper and some others.

Of course, nothing new is presented in this very brief communication except that greater confidence in this plan of treatment may be imparted by the naked statement of facts as above made.

The restraints of adhesive plasters, with cord, pulley and weights, are much less than with the older treatment by means of splints, and hence much more tolerable. Moreover the arrangement of pulley and weights approximates that of the simpler treatment with pillows, &c., nevertheless, the smallest restraints may fret the patient, and militate against success, and if we learn from our own experience and the testimony of others, that good results are apt to follow the trusting of the fracture to time and nature, we shall be more prompt in resorting to such a course.

In concluding, I may relate one interesting case and rather remarkable in the fact that eight years after recovering from a fracture on one side, and using very comfortably, a cork sole $3\frac{1}{2}$ inches thick, and walking a mile to churh, she fell over a carpet and fractured the other femur in the same place. She recovered with the same simple treatment, and now both legs are of the same length and she walks as well as before. This lady is now 76 years of age; the last fracture was two years ago.

Yours Truly, SAM'L PETERS, M. D.

CRESCENT, N. Y., October 24, 1873.

